# Decision making in the end-of-life care of patients who are terminally ill with cancer – a qualitative descriptive study with a phenomenological approach from the experience of healthcare workers

**DOI:** 10.1186/s12904-021-00768-5

**Published:** 2021-05-28

**Authors:** Angela Luna-Meza, Natalia Godoy-Casasbuenas, José Andrés Calvache, Eduardo Díaz-Amado, Fritz E. Gempeler Rueda, Olga Morales, Fabian Leal, Carlos Gómez-Restrepo, Esther de Vries

**Affiliations:** 1 Department of Clinical Epidemiology and Biostatistics, Faculty of Medicine, Pontificia Universidad Javeriana, Bogota, Colombia; 2 Internal Medicine Resident, Faculty of Medicine, Pontificia Universidad Javeriana, Bogota, Colombia; 3 PhD Programme in Clinical Epidemiology, Department of Clinical Epidemiology and Biostatistics, Faculty of Medicine, Pontificia Universidad Javeriana, Bogota, Colombia; 4 Department of Anesthesiology, Universidad del Cauca, Popayán, Colombia; 5 Department of Anesthesiology, Erasmus University MC Rotterdam, Rotterdam, The Netherlands; 6 Institute of Bioethics, Pontificia Universidad Javeriana, Bogota, Colombia; 7 Faculty of Medicine, Pontificia Universidad Javeriana, Bogota, Colombia; 8 Hospital Universitario San Ignacio, Bogota, Colombia; 9 Servicio De Dolor y Cuidados Paliativos, Department of Anesthestiology, Faculty of Medicine, Pontificia Universidad Javeriana, Bogota, Colombia; 10 Instituto Nacional de Cancerología, Bogota, Colombia

**Keywords:** Neoplasms, Terminal care, Palliative care, Death, Colombia

## Abstract

**Background:**

In Colombia, recent legislation regarding end-of-life decisions includes palliative sedation, advance directives and euthanasia. We analysed which aspects influence health professionals´ decisions regarding end-of-life medical decisions and care for cancer patients.

**Methods:**

Qualitative descriptive–exploratory study based on phenomenology using semi-structured interviews. We interviewed 28 oncologists, palliative care specialists, general practitioners and nurses from three major Colombian institutions, all involved in end-of-life care of cancer patients: Hospital Universitario San Ignacio and Instituto Nacional de Cancerología in Bogotá and Hospital Universitario San José in Popayan.

**Results:**

When making decisions regarding end-of-life care, professionals consider: 1. Patient’s clinical condition, cultural and social context, in particular treating indigenous patients requires special skills. 2. Professional skills and expertise: training in palliative care and experience in discussing end-of-life options and fear of legal consequences. Physicians indicate that many patients deny their imminent death which hampers shared decision-making and conversations. They mention frequent ambiguity regarding who initiates conversations regarding end-of-life decisions with patients and who finally takes decisions. Patients rarely initiate such conversations and the professionals normally do not ask patients directly for their preferences. Fear of confrontation with family members and lawsuits leads healthcare workers to carry out interventions such as initiating artificial feeding techniques and cardiopulmonary resuscitation, even in the absence of expected benefits. The opinions regarding the acceptability of palliative sedation, euthanasia and use of medications to accelerate death without the patients´ explicit request vary greatly. 3. Conditions of the insurance system: limitations exist in the offer of oncology and palliative care services for important proportions of the Colombian population. Colombians have access to opioid medications, barriers to their application are largely in delivery by the health system, the requirement of trained personnel for intravenous administration and ambulatory and home care plans which in Colombia are rare.

**Conclusions:**

To improve end-of-life decision making, Colombian healthcare workers and patients need to openly discuss wishes, needs and care options and prepare caregivers. Promotion of palliative care education and development of palliative care centres and home care plans is necessary to facilitate access to end-of-life care. Patients and caregivers’ perspectives are needed to complement physicians’ perceptions and practices.

**Supplementary Information:**

The online version contains supplementary material available at 10.1186/s12904-021-00768-5.

## Key message

The results highlight the importance of improving access to end-of-life care in Colombia, and diminishing the “denial of imminent death” among patients and caregivers to facilitate end-of-life discussions and shared decisions; interventions to prepare caregivers and promote home care.

## Introduction

Despite advances in prevention, early diagnosis and new treatment options, many cancer patients eventually develop a terminal and advanced stage of disease, generating situations in which decision-making is difficult for patients, caregivers and doctors [1].

End-of-life (EoL) care, as part of palliative care, revolves around maintaining the quality of life of patients and their families, through comprehensive management of physical symptoms, psycho-social and spiritual difficulties. End-of-life care focuses on a short period of time before death [2] and includes discussion of medical practices (suspending or withholding specific cancer treatment and general care related to feeding, hydration, and resuscitation techniques; symptom management; euthanasia) and decision-making [3, 4]. These decisions include p*alliative sedation* [5] and euthanasia.

Colombia is a middle-income country in South America, with a population around 49 million people. It is progressing to reach the objectives proposed by the World Health Organization (WHO) regarding palliative care [6].

In 2014 the Colombian government introduced “Consuelo Devis Saavedra Law” (Law 1733), aiming to integrate palliative care within public health programs and regulate palliative care services for the comprehensive management of patients with terminal, chronic, degenerative and irreversible diseases, guaranteeing patients the right to receive palliative care as well as the right to voluntarily desist from medical treatments that they consider unnecessary [7]. From 2014 onward, multiple documents and treatment guides have been issued, promoting adequate availability of opioids, the development of more health care services dedicated to palliative care and creation of more educational programmes [8].

A study that estimated palliative care needs for oncological and non-oncological diseases in Colombia, documented that by 2016, 61% of deaths in the country were related to causes that would probably require palliative care, of these 31.3% were related to cancer. In the same study, when comparing the need for palliative care with the formally available offer of these services, it is evident that in some regions of the country there is no offer at all and in other high-need areas the offer is limited [9].

Colombia is the only country in Latin America with a regulation to apply euthanasia. Although the Colombian Constitutional Court decriminalized “mercy killing” in 1997, it was not until April 2015 that the Ministry of Health specified how it could occur. Currently, the practice must be guided by the Protocol for the application of the euthanasia procedure in Colombia 2015 [10, 11].

Advance-care conversations and directives are increasingly used in high-income settings, seem to improve quality of life and quality of dying, and reduce unnecessary treatments [12–14].

There are some clinical and technical criteria to guide decisions at the EoL, but the patient’s situation around the time of death is so particular to personal circumstances that decisions are mostly guided by clinical judgement and prior expertise [15].

Studies considering medical decision-making at the EoL have been mainly conducted in Europe the United States and Australia [15–18]. No such studies have been conducted in Latin America. As cultural and social views on death vary greatly, and Colombia recently regulated euthanasia, it is important to explore what aspects determine decision-making at the EoL to identify difficulties and promote strategies to facilitate conversations, advance care planning and decision-making.

## Methods

### Design

We conducted a qualitative descriptive-exploratory study based on phenomenology [19] to explore the experiences of healthcare workers in decision-making regarding EoL care of cancer patients through individual face-to-face semi-structured interviews. This approach recognizes that the experience of physicians making EoL decisions incorporates the interaction between professionals and their patient as well as their personal history and medical education and the influence of the health care system.

### Participants and setting

This study was conducted in three Colombian institutions: Hospital Universitario San Ignacio and Instituto Nacional de Cancerología, both in Bogota (capital city), and Hospital Universitario San Jose in Popayan, located in the southwest of Colombia attending urban and rural populations. Hospital Universitario San Ignacio has a specialized centre for oncology and palliative care and is mostly attended by residents from Bogota (capital), most of whom are insured by health insurance paid on the basis of a percentage of their income (contributive insurance), which implies they have income. The Instituto Nacional de Cancerología is attended by 10% of all Colombian cancer patients, from a variety of socioeconomic background and health insurance, most patients are from the region close to Bogota, but an important proportion of patients come from remote areas. The population who attend Hospital Universitario San Jose is largely of rural origin, living far away from big cities and clinics, many of whom are from indigenous communities, most patients having a government subsidized health insurance type – implying low income.

Purposive sampling was employed to identify healthcare professionals who provide medical care for cancer patients at the EoL, including oncologists, pain and palliative care specialists, general practitioners and nurses that worked in these areas. Selection of participants was carried out seeking maximum diversity of the sample including, gender, time of experience with terminally ill patients, and socioeconomic conditions of patients they provide care for. The research coordinator in each of the three participating hospitals provided names of the professionals matching the profiles.

### Ethics approval and consent

The study procedure was approved by the research ethics committees at Instituto Nacional de Cancerología (number INT-OFI-03581-2019) and Pontificia Universidad Javeriana (number FM-CIE-0086-17). Hospital Universitario San Jose accepted the latter. All participants signed informed consent.

### Data collection

The interviewer (A.L., female general physician, masters in clinical epidemiology student and internal medicine resident, with no formal relationship to the participants) collected informed consent and sociodemographic data of the participants after which she proceeded to the interview, based on an interview guide (supplementary Table 1). The interview guide was previously pilot tested, and included questions about participants’ perspectives on the influence of the clinical and socio-cultural condition of patients, the doctor-patient relationship, conditions of the health system, and ethical concerns in the decision-making process at the EoL.

Interviews were conducted between November 2018 and July 2019. One invited physician refused to participate due to lack of time. Interviews were done at the workplace, and audio recorded. Each interview lasted approximately 30–40 min. All personal information was anonymized for analysis and reporting.

### Data analysis

A qualitative analysis of semi-structured interviews was conducted, coding was performed by two researchers (A.L. and N.G.), using a phenomenological approach and discourse analysis [20–22]. Interviews were transcribed verbatim and coded using NVivo 12 software. Segments of transcripts were assigned codes based on their significance. Emergent codes were revised through discussions within the research team. Through an inductive process, examining significance, finding similarities and relationships, codes were organized into categories and later into themes. Initially, a total of 20 interviews were planned. Though there is not a definitive test for data saturation, according to the methods proposed by Francis et al., we defined that when the research team were confident that saturation had been reached an additional three interviews were carried out to make certain no new themes arose [23]. Following this method interviews were performed until three consecutive interviews with data saturation were obtained, resulting in a total of 28 interviews.

## Results

Twenty-eight semi-structured interviews were conducted. Demographic characteristics of participants are shown in Table 1. The mean age of participants was 37.4 years (ranging from 23 to 59), 82% were physicians (*n* = 23) and 75% (*n* = 21) worked in palliative care settings.

**Table 1:** Demographic characteristics of participating healthcare professionals (*n* = 28)

Variables	n (%) or mean (SD)
**Sex**	
Female	10 (36%)
Male	18 (64%)
**Age in years (mean, SD)**	37.4 (9.6)
**Race/Ethnicity**	
White	6 (21%)
Mestizo^*a*^	20 (71%)
Black	0 (0%)
Indigenous	1 (3%)
Other	1 (3%)
**Education**	
Medical oncologist	6 (21%)
Palliative care physician	9 (32%)
Anaesthesiologist	1 (4%)
Internal medicine physician	1 (4(%)
General practitioner	6 (21%)
Nurse	2 (7%)
Nurse assistant	3 (11%)
**Discipline:**	
Oncology	7 (25%)
Palliative care	21 (75%)
**Years of practice with cancer patients at the end-of-life**	
Less than 6 months	0 (0%)
6 to 12 months	1 (3%)
1 to 4 years	12 (43%)
More than 4 years	15 (53%)

*SD* standard deviation

^*a*^ Mix of white and indigenous race

Through the interviews three main themes emerged around decision-making and medical practices in the EoL care of patients who are terminally ill with cancer:

1. The cancer patient from the health professionals’ point of view
2. Physicians’ skills and expertise
3. Medical practices at the EoL and their application in the health system

Throughout the results, textual citations that justify analysis are presented. In order to give context to the reader, each quote is referenced to the profession of the person who said it, maintaining anonymity, as follows: oncology specialist (Onc), pain or palliative care specialists (Pall), general practitioner who works in these areas (GP) or nursing staff (Nur).

### The cancer patient from the health professionals’ point of view

#### Physical needs

Most participants describe patients predominantly from the perspective of the disease: the symptoms and functional impairment due to the illness. The main source of concern identified for patients, families, and participants themselves was symptom-related suffering, most importantly pain.

*"I think the most frequent concerns are the physical symptoms: pain, thirst, respiratory distress” Onc.*

#### Non-organic needs

Some health-professionals described patients’ feelings and other sources of concern such as spiritual aspects, family, and financial matters. Also, most physicians mentioned patients may be afraid of being “abandoned” at the EoL, especially when disease-modifying therapy such as chemotherapy is no longer an option.

*“Most patients, especially those that support their family are concerned about their financial needs during illness and after they’ve passed away, saying -after I die who is going to take care of my family?-” Pall.*

Interviewees described how for patients living in rural areas, the progression of their disease to end of life, is not only determined by the course of the illness but also by limitations to receive appropriate treatment due to distances and economic implications of displacement to the city.

*“(…) In Popayan and probably in Colombia in general, one of the main barriers is the need to go to different cities for treatment, some patients travel 2 or 3 hours on horseback (…) they miss appointments because they don’t have money (…) ” Pall.*

*“Some (patients) say: - I don’t live here. If I die here (in the city), taking my body home will be expensive. They live very far and are poor” Nur.*

In Popayan, patient’s culture takes on greater relevance, especially for patients belonging to indigenous populations. They have a different, more peaceful vision of death and their beliefs and traditions expand management options to include traditional medicine.

*“When discussing the end of life with indigenous people the situation changes dramatically. Both the cultural and religious perception of death is completely different. People who have ancestral beliefs live the process of death and dying in a more peaceful way” Pall.*

*“I do not debate his (the patients’) beliefs or his attitude towards end of life. I try to adapt and for that I have tried to educate myself regarding their cultural and historical aspects, about the way they think and feel, and what might be important to them” Pall.*

#### Caregivers concerns

Participants identified patients as autonomous individuals with the right to be informed and make decisions regarding their health. Later in the course of their disease, when perhaps the level of conscience of the patient is affected, the family usually act as the patient’s representative.

Families residing in rural areas are identified as generally more willing to take care of the patients at home. They consider pain, suffering and death as processes that are a part of life, and are familiar with an active role that families and communities take in caring for people. Economic implications of transportation of a deceased patient for a funeral, should the patient die in a different city, may also influence this willingness. Participants indicated that family members who are reluctant to take care of patients at home feel unprepared and have less time and economic resources available to be able to provide the needed care to their relative.

*"when the family does not have the means to provide the necessary comforts at home to take care of the patient, they prefer he’d stay at the hospital” Nur.*

*“country people have greater support of the family to take care of them at home” Pall.*

### Physicians’ skills and expertise

*The doctor-patient relationship.* For health-professionals, the oncologist has the main responsibility for cancer patients: providing cancer treatment, giving information regarding disease progression and prognosis, and making initial EoL decisions. Patients often don’t have access to palliative care from an early stage in the disease as a consequence of the way the Colombian healthcare system works and because of a shortage of palliative care services. Where oncology services are not available, palliative care, internal medicine specialist and surgeons are identified as those in charge of EoL decisions.

*“(...) cancer patients have a strong bond with their oncologist. The health care system has left it to us, most doctors and health professional tell them -ask your oncologist, what does your oncologist say?-. Patients trust us a lot.” Onc*

Oncologists report that in each visit they inform patients and caregivers in relation to the diagnosis, prognosis and the intentions or aims of the treatments the patients are receiving. Yet, most healthcare professionals mention that patients frequently state that they were not informed regarding their disease status. Professionals interpret this apparent discrepancy, between physicians claiming to inform patients and patients stating not to be informed, to the patients being in a state of denial within the mourning process of having a terminal disease.

*"… many patients say: -nobody told me anything-, but this is part of the process of denial, it’s completely normal (...), that is why we reinforce our concepts in the medical records” Onc.*

Empathy (ability to recognize and understand patients’ emotions) and trust are the two main attributes identified by participants in the doctor-patient relationship, both developed through communication. Participants mention that special communication skills are required to give bad news and communicate EoL options and decisions. This skill set is required to provide clear information without eliminating hope in the patient while also conveying confidence from the physicians’ point of view. Participants highlighted that this skill is acquired through experience.

*"I believe that the most important attributes of the doctor-patient relationship are empathy and trust" Onc.*

Some physicians are identified by colleagues as people with greater empathic ability to communicate with patients at EoL. Upon interviewing these physicians, we found they had more experience working with patients at the EoL and all talked with their patients about non-organic needs and worries. They expressed that time invested in these conversations leads to greater knowledge of the patient’s life, builds trust, opens a comfortable space for patients to express their own ideas regarding management options and initiate EoL discussions.

*“Over time, I inadvertently learn some things about his (the patient) life: e.g. what his children do, and that makes him feel important, he trusts me.” Onc.*

Training in end-of-life care is considered insufficient and theoretical. Physicians consider communications skills to inform and discuss end-of-life management options are skills acquired through practice and experience.

*“I am convinced that communicating appropriately requires sensitivity. One can train [in] that and become proficient in the ability to inform in a smooth but realistic way the patient’s situation and the possible outcomes” Pall.*

### Medical practices at the end of life and their application in the health system

The Colombian healthcare system is a barrier to a trusting patient-doctor relationship. Cancer patients are frequently forced to change healthcare provider because of changing contracts between their health insurance and providers. This move from one physician to the next may result in the patient and their family perceiving the treating physician at the EoL as a stranger who has come to diminish hopes and expectations.

*“When I see the patient for the first time and he is already at the very last phase of life, it’s more complicated. If I’m presenting palliative sedation as an option or talking about home-care options, for example, there will be mistrust of the patient and the family. Many times, there are confrontations. They don't want the participation of an unknown person who is also cutting their hopes and expectations" Pall.*

Decisions made about EoL care involve changes in the treatment and practices related to dying:

*Not initiating or suspending specific cancer treatment* (mostly chemotherapy) is a decision made by the oncologist who considers the patient’s clinical condition, functional and nutritional status as the basis to contemplate treatment options. Participants explained that it is always more difficult to suspend treatments than to not initiate them, and although there are some clinical criteria to guide these decisions, clinical judgement remains key.

*"The difficult part of oncology is not deciding when to start a treatment but to know when to stop it, (…) the decision to stop is related to the functional status of the patient and his/her prognosis and treatment goals” Onc.*

Earlier referral and availability of palliative care services would diminish patient’s fear of abandonment and facilitate decision-making. In Popayan, very limited access to oncology services sometimes implied that palliative care is the main attention received by cancer patients from diagnosis, representing frustration for all involved.

*"Many times, patients keep going to the oncologist until death because they are afraid no one will take care of them anymore. If they know that we’ll be the ones (palliative care team) who take care of them until death and even after that - care for the family is part of our grief protocol - it is easier to stop specific cancer treatment and other interventions” Pall.*

#### Suspending general care measures

This practice most frequently presented ethical dilemmas for the participants. This was particularly focused on who should make these decisions and who should start EoL conversations with the patients? There was no clarity among professionals how these roles are and should be distributed.

Healthcare workers identify nutrition as the main source of anxiety at EoL among patients and caregivers. As long as the patient can eat, intake is guided by patients’ wishes. Oncologists are generally against artificial feeding techniques (enteral and parenteral feeding), other physicians argue these may present possible benefits in terms of quality of life. All participants agreed that once artificial feeding techniques have been established, withdrawing these is almost impossible: this causes ethical dilemmas and confrontation with patients and caregivers.

Participants also agreed that artificial hydration, when provided in a home-care setting, requires training, delivery of resources and availability of programs to avoid and manage complications. Oxygen is considered a basic measure and hardly ever withdrawn as this generates feelings of abandonment.

*“To decide to stop nutrition, oxygen is much more difficult. It is more ethical to say from the start it has no indication; we are not going to give it . But when the patient already has a gastrostomy, or a tracheostomy, I cannot say ‘take it off’, it is a situation we do not want to reach. It is more ethical not to start them, than to have to withdraw them later” Pall.*

Regarding advanced procedures such cardiopulmonary resuscitation procedures, participants stated that patient wishes should guide decisions, but that patients hardly ever have advance directives. Some physicians think patients do not initiate EoL conversations because they do not want to talk about death. Despite this, few health-professionals ask patients directly for their preferences and state that most patients do not express clear wishes.

*"It’s not common for us to talk about death. We talk about symptom control, patients know that their disease has progressed, and that death is near, but they almost never ask questions about it and for me to bring it up, it feels like putting my finger deeply into this wound. So, I ask indirect questions like-‘where do you want to be now that we are going to de-escalate the treatment?’ ” Onc.*

Some physicians considered “do not resuscitate” agreements are valid medical options in this scenario. Others discussed that fear of confrontation with family members and lawsuits can lead doctors to carrying out interventions ranging from initiating artificial feeding techniques to initiating cardiopulmonary resuscitation, even in absence of expected benefits.

*“When patients are taken to the emergency department because of dyspnoea some family members say ‘I want him to be resuscitated, to be intubated, to do everything possible’ and it is difficult for the doctor to go against that wish, especially from a legal point of view, the doctor usually ends up performing these manoeuvres, even when, in his opinion, it is useless” Pall.*

#### Intensified symptom management

All participants considered this practice the basis of EoL care. The existence of opioid medications with different relative potency and routes of administration available in Colombia was one of the strengths mentioned. Barriers to their application are largely because of difficulties in delivery by the health system (lack of consistent availability as well as health insurance companies sometimes denying the patients their medication), the requirement of trained personnel for intravenous administration and ambulatory and home care plans which in Colombia are rare.

*"Let's say a patient requires a subcutaneous morphine infusion. That is relatively simple. But for us there is no way to do it at home. So, it would be a waste of time to inform the patient about this option of home-care, since in the end it won’t be possible. I do believe that we are still lacking home-care services throughout the country and particularly in Popayan” Pall.*

#### Palliative sedation

Oncologists had a clear definition of palliative sedation but considered this beyond their area of expertise. Most physicians working in palliative care had a clear concept of this practice, they commonly consider in-hospital settings and require in most situations a written consent of the patient or caregiver. Some health-professionals considered that this practice can accelerate death and therefore have ethical objections.

*"It is what we call the double effect principle, I give a medication to improve a symptom that has become intolerable, but as a secondary effect, having a decrease in consciousness can lead to ventilatory failure, that is explained and it is written as such in the authorization that they (patient and/or family) must sign. That is what they (patient and family) are most afraid of, but we explain to them (...) sedating or not sedating does not make a significant difference in the time of death, and given that the patient is already at the end of his life , what matters is quality of life and death " Pall.*

#### Euthanasia

Participants explained they very rarely receive requests for euthanasia. Participants explained that they considered euthanasia as not yet culturally accepted and therefore their patients do not request information. Additionally participants highlighted that they do not feel prepared to discuss euthanasia as there remains a lack clarity regarding procedures.

Participants outlined their belief that euthanasia should not be mentioned as an option before a patient mentions it themself; this would be considered to go against the principles of professional practice.

*"(Euthanasia) is not common here, it seems to not be culturally accepted, occasionally a patient has asked about it but not with the legal formality needed… It seems to me that patients don’t know much about this practice” Nur.*

One physician believed that some of his patients may have committed suicide and thought these patients may have considered but never formally requested euthanasia or had difficulties in obtaining approval. Another physician considered that using medications to accelerate the moment of death of patients in the final stages of life can be a valid medical decision to “facilitate a dignified death”, even without the request or consent of patients or relatives.

All participating palliative care physicians were against euthanasia arguing that optimal palliative care is still not accessible to all patients in Colombia and therefore euthanasia should not “replace” optimal care. They also related euthanasia with a feeling of failure and frustration for not being capable to control patients’ symptoms. Some considered the approved protocol for euthanasia in Colombia technically inadequate, for others euthanasia went against their personal religious beliefs.

*“The philosophy of palliative care does not favour assisted suicide or euthanasia. Our aim is for the patient to live what they have to live but without suffering, we understand death as a natural process and we highly value the lives of patients, we consider human life has an intrinsic value” Pall.*

## Discussion

### Main findings

This study explored perspectives of health-professionals on the decision-making process in EoL care of terminally ill cancer patients in different settings in Colombia. Three main conditions influence this process: patients clinical and socio-cultural conditions, physician’s relationship with patients and caregivers and palliative care education, and health system possibilities and limitations.

Participants mention that the factors which most influence decisions have to do with the expected benefit of each intervention alleviating suffering and whether or not the interventions will improve quality of life – which is in line with the literature [24].

The plurality of cultures within the Colombian population causes frequent differences in the healthcare worker’s culture versus their patient’s culture, requiring special skills in management of any diversity which may exist in relation to this. Healthcare workers caring for patients from rural areas must consider distance from treatment centres, economic limitations for traveling and limited access to home-care services in the decision-making process, as has been reported elsewhere [25, 26].

Of note, in a recent systematic review regarding rural EoL care from the experiences and perspectives of patients and family caregivers reported the most important need was related to receive accurate information and clear communication from healthcare workers both for developed and developing countries, as well as the need for pain relief and access to basic medicatios in sub-Saharan Africa [25]. This review did not include any study from Latin America. Their findings largely coincide with the experience of our participants, with a particularity from our setting being the comments on the many patients coming from rural areas who are part of indigenous populations. Our participants indicated that when patients belong to indigenous groups, physicians are required to understand patients’ beliefs, the structure and functioning of families and communities as a social organization, be open to some alternative management options from traditional medicine and have enough time to discuss each of these topics.

Previous investigations from patients’ perspectives have shown the main problems in relation to end-of-life care are communication, difficulties in accessibility and responding quickly to acute problems [27]. In the main cultural groups in Colombia, there is taboo in relation to talking about death and dying. Results from our study highlight healthcare workers’ perceptions that patients are not willing to talk about these topics and this impedes optimal decision-making processes, leading to suboptimal communication and potentially suboptimal care and symptom management [28, 29]. Unfortunately, there are no studies from the patients’ perspectives from similar populations to contrast these findings against. It seems that in Latin America many physicians may feel their task is to cure patients, and that accompanying them to a “good death” is not their responsibility. Nevertheless, these findings are in agreement with studies in other international populations where talking openly about death and dying is not acceptable because it is considered disrespectful, bad luck, or causes loss of hope [30–32].

Important barriers to proposing home-care for patients in EoL, besides fear of abandonment by the healthcare system among patients and caregivers [33], centre on the very limited offer of home-based palliative care and perceived feelings of unpreparedness from caregivers [34, 35]. Unfortunately, there are no studies from the caregivers´ perspective in our population to contrast physicians’ impressions. In addition, regulation regarding prescription, delivery and application of opioids hamper home-care of patients; professionals are required to formulate most drugs and delayed delivery causes a barrier which is not always necessary [29, 36]. In other LMIC home-care including effective opioid administration, from prescription to administration, has been made possible [37, 38].

The literature describes lack of clarity as a significant barrier for doctors in decision-making during the end of life [39]. Our participants identified these same problems, but in addition mentioned feelings of frustration, confrontation with caregivers and fear of lawsuits as barriers. For palliative sedation and euthanasia to be accepted, having appropriate regulation is not enough. It is also necessary to socialize the requirements, processes, and protocols with patients and healthcare workers, since confusion regarding these topics cause ethical dilemmas and limited discussions and, in some particular scenarios, might generate situations where drugs are applied to terminate the lives of patients even without their request or consent. On the other hand, our data shows that perception of lack of openness to discuss euthanasia may lead to patients committing suicide.

There is overlap in our findings in relation to: the importance of the expected benefit of each intervention alleviating suffering and improving quality of life; and difficulties in communication between physicians and patients. These are reflective of findings from other countries [23–25, 30–32]. However others, like lack of access to early palliative care and fragmentation of care in different institutions and cities, have not been reported from other countries and have to do with the socio-cultural circumstances in Colombia combined with limited healthcare system capabilities [40].

### Strengths and limitations of the study

The strengths of this first study to report the situation in Latin America include the large and diverse group of participants, receiving comprehensive responses on the most controversial topics and having achieved data saturation. The participating institutions represent different social and cultural contexts, although the selection of participants mainly from major hospitals may not represent the reality of several more rural areas of the country where patients are mainly cared for by general practitioners. It would be interesting to include more testimonies from other health-professionals. Another limitation was the difficulty in examining more closely ethical dilemmas related to EoL care identified by healthcare workers and their ability to analyse and resolve them. Some problems of the health system that lead to difficulties described in the decision-making process, such as availability and rapid authorisation of treatment by health insurances, geographical and travel distances, deserve more in-depth research.

### Implications for practice and research

Further research on patients’ thoughts about the decision-making process at EoL in Colombia is necessary to contrast our healthcare workers’ perceptions and to develop strategies that facilitate starting EoL conversations. It is necessary to promote further education of health-professionals in the practical and ethical issues and application of end-of-life care and in other social and cultural aspects of the populations that have direct implications on the final stage of life. Also, there is a need to inquire about caregivers' concerns and design programs to train and support them in the provision of EoL care.

It is essential to promote continuity in EoL care delivery by a fixed, skilled and stable team of health-professionals as this facilitates a trusting doctor-patient relationship and allows more time to discuss topics different to clinical needs.

More home-based care services and palliative care centres combined with effective communication between them and oncology services are required to facilitate access to specialized care and transition to EoL care.

## Supplementary Information

**Additional file 1: Supplementary Table 1.** Interview guide.

## Data Availability

All data sets on which the conclusions of the paper are based are available upon request to the corresponding author.

## Abbreviations

EoL: End-of-Life
QoL: Quality of Life
