# Supplementary material for: Decision making in the end-of-life care of patients who are terminally ill with cancer – a qualitative descriptive study with a phenomenological approach from the experience of healthcare workers
Source: BMC Palliat Care. 2021 May 28;20:76. doi: 10.1186/s12904-021-00768-5 (PMC8164310; doi:10.1186/s12904-021-00768-5)
Supplement: Supplementary file 1 — Additional file 1: Supplementary Table 1. Interview guide. [file 12904_2021_768_MOESM1_ESM.pdf]

Decision making in the end-of-life care of patients who are terminally ill with cancer – a qualitative descriptive study with a phenomenological approach from the experience of healthcare workers.

Angela Luna-Meza, Natalia Godoy-Casasbuenas, José Andrés Calvache, Eduardo Diaz, Fritz Gempeler, Olga Morales, Fabian Leal, Carlos Gómez-Restrepo, Esther de Vries

Supplementary table 1. Interview guide

What is it like to tell someone that his/her life is ending?
Where do you prefer this conversation to happen?
When informing the patient, what management options can be offered?
Of these practices proposed in literature, which are put into practice in the Colombia?<br>• Do not initiate or suspend cancer-specific treatments<br>• Do not initiate or suspend general management measures<br>• Intensify symptom management<br>• Palliative sedation<br>• Assisted suicide<br>• Euthanasia
Do you think your professional training has given you enough tools to end-of-life discussions and decision-making?
How would you describe a terminally ill cancer patient?
What is it like to make end-of-life care decisions with cancer patients?
